# AMPA Receptor Modulation Through Medium-Chain Triglycerides and Decanoic Acid Supports Nutritional Intervention in Pediatric Epilepsy

**DOI:** 10.3390/nu17111805

**Published:** 2025-05-26

**Authors:** Raffaele Falsaperla, Vincenzo Sortino, Miguel Angel Soler, Michela Spatuzza, Sara Fortuna, Vincenzo Salpietro

**Affiliations:** 1 Department of Medical Science-Pediatrics, University of Ferrara, 44124 Ferrara, Italy; 2 Pediatrics and Pediatric Emergency Department, Azienda Ospedaliero-Universitaria Policlinico “Rodolico-San Marco”, San Marco Hospital, 95121 Catania, Italy; sortino.vinci@gmail.com; 3 National Council of Research, Institute for Research and Biomedical Innovation (IRIB), Unit of Catania, 95126 Catania, Italy; 4 Dipartimento di Scienze Matematiche, Informatiche e Fisiche, Università di Udine, 33100 Udine, Italy; miguelangel.solerbastida@uniud.it; 5 Italian Institute of Technology (IIT), Via Melen 83, B Block, 16163 Genova, Italy; fortuna.sara@gmail.com; 6 Department of Biotechnological and Applied Clinical Sciences, University of L’Aquila, 67100 L’Aquila, Italy

**Keywords:** MCTs, AMPA, drug-resistant epilepsy, ketogenic diet

## Abstract

**Background:** Developmental epileptic encephalopathies (DEEs) are often associated with variably severe cognitive and motor impairment and frequent refractory epilepsy, with many children not achieving adequate seizure control via standard antiepileptic medications. The classic ketogenic diet (KD) has proven effective in reducing seizure frequency and/or severity in a category of DEEs and in certain refractory epilepsies of infancy. However, its multifaceted mechanisms, e.g., epigenetic modulation, anti-inflammatory and antioxidative effects, and direct neuronal excitability changes, are balanced by a high burden and low long-term adherence. Medium-chain triglycerides (MCTs), particularly decanoic acid (C10:0), have gained attention in recent years for their potential direct inhibitory action on AMPA receptors, contributing to seizure reduction. **Methods**: A systematic review was conducted, including articles from January 2000 to January 2025, to explore the potential role of medium-chain triglyceride (MCT) add-on to classic KD and as MCT supplementation in free diets in the management of pediatric drug-resistant epilepsy (DRE). **Results**: Selected studies show how the action of MCTs, and decanoic acid in particular, is via negative modulation of AMPA receptors, with a positive impact on epileptic seizures. **Conclusions**: This review discusses the complexities of implementing and sustaining KD in children and presents recent pre-clinical and clinical evidence, including trials where MCTs (often enriched in decanoic acid) serve as an add-on therapy in both ketogenic and free/unrestricted diets. The summarized findings reinforce the therapeutic potential of MCTs, highlighting both the beneficial seizure outcomes and the hurdles that remain to be addressed through future research.

## 1. Introduction

For children living with drug-resistant epilepsy (DRE) and their families, daily life can be compromised by the unpredictability and severity of frequent seizures. These children often undergo multiple trials of antiepileptic drugs (AEDs) yet remain vulnerable to ongoing seizure activity that impairs their safety, neurodevelopment, and overall quality of life [1]. These challenges impact not only the children but also their caregivers, who suffer significant emotional and logistical burdens while seeking effective therapies [2].

In recent years, dietary interventions have emerged as promising adjuncts to help fill this therapeutic gap. Chief among these is the ketogenic diet (KD), a high-fat, low-carbohydrate regimen that fundamentally shifts the body’s metabolism toward ketone body production. Beyond anecdotal reports, robust clinical evidence shows that KDs can substantially reduce seizure frequency in a subset of children [3]. Proposed mechanisms include epigenetic regulation—where ketones can influence gene expression affecting neuronal excitability—along with anti-inflammatory and antioxidative effects that protect neural tissues, and direct synaptic and metabolic modulation that alters ion channel function and neurotransmitter balance [4].

Despite these scientific and clinical results, families may find it challenging to initiate and maintain such a strict dietary protocol. Consequently, poor long-term adherence is a common stumbling block [5].

An increasingly explored alternative is the use of medium-chain triglycerides (MCTs), particularly decanoic acid (C10:0). Research suggests that decanoic acid can directly inhibit AMPA receptors, a key component in excitatory glutamatergic signaling, thereby easing the neuronal hyperexcitability that underlies many seizure disorders. Notably, these MCTs may be used in two ways: either to enhance the effectiveness of a classic ketogenic diet, or as a dietary supplement added to a more flexible or unrestricted diet. [6,7,8].

This review aims to present both preclinical and clinical data on MCT use and the broader KD including both adult and pediatric patients, paying particular attention to the latest evidence on how decanoic acid may work to modulate AMPA receptors.

## 2. Materials and Methods

This systematic review was conducted according to PRISMA guidelines to enhance transparency and methodological rigor. Our objective was to explore the use of MCTs, particularly decanoic acid, in KD therapies for DRE, with a focus on pediatric populations.

### 2.1. Search Strategy and Eligibility Criteria

- Database Searched: The search was performed on PubMed, and the last search was carried out on 31 January 2025. The keywords used included: “ketogenic diet”, “medium-chain triglycerides”, “decanoic acid”, “AMPA receptor”, “drug-resistant epilepsy”, “pediatric epilepsy”, and “MCT supplementation”. The systematic literature search was conducted employing the Boolean operator AND to combine pairs of selected keywords, thereby restricting the results to studies containing all specified terms within each query. As part of the search strategy, in accordance with PRISMA guidelines, we applied predefined filters on PubMed to restrict results based on the inclusion criteria. These filters included publication type (e.g., original articles, clinical studies, RCT etc.), language (English), and publication date range to ensure that only studies relevant to the objectives of the systematic review were considered.
- Study Period: We included articles published from January 2000 to January 2025 in order to collect the latest scientific evidence.
- Inclusion Criteria:
  1. Preclinical Studies: Investigations using cellular/in vitro models or animal models that assessed seizure thresholds, seizure frequency, receptor-binding characteristics, or relevant mechanistic insights tied to MCTs or decanoic acid.
  2. Clinical Studies: Research on pediatric DRE or adults where mechanistic insights are relevant to pediatrics (including observational studies, case series, pilot/feasibility studies, randomized controlled trials (RCTs), and meta-analyses).
  3. AMPA Receptor Modulation Mechanisms: Studies that provided mechanistic or functional data on the interaction between decanoic acid (or MCTs) and excitatory glutamatergic pathways.
  4. Articles in English.
- Exclusion Criteria:
  1. Reviews, editorials, and conference abstracts without original data.
  2. Studies focused exclusively on adult epilepsy or other pathologies without translational relevance.
  3. Studies unrelated to MCTs or ketogenic diets.

### 2.2. Study Selection and Data Extraction

After removing duplicates, two reviewers independently screened the article titles and abstracts for relevance. Full-text articles were retrieved for those deemed potentially eligible or when abstracts provided insufficient information. Discrepancies in inclusion/exclusion were resolved through discussion. The key data extracted included:

- Study Type and Design: Preclinical (in vitro, animal), observational (retrospective/prospective cohorts), RCT, meta-analysis.
- Population: Age range, epilepsy etiology/subtypes, DRE status.
- Dietary Intervention: Classic KD, MCT-based KD, or free diet with MCT supplementation; specific dosing regimens if provided.
- Endpoints: Seizure frequency/severity, metabolic biomarkers (ketone levels, lipid profiles), tolerability/adverse events, adherence rates, neurocognitive outcomes, and putative mechanistic measures (e.g., receptor binding, neuroinflammatory markers).
- Results: Efficacy measures (e.g., % seizure reduction, seizure-freedom rates), mechanistic findings (AMPA receptor modulation), and study limitations.

### 2.3. Data Synthesis and Analysis

Given the heterogeneity in study populations, dietary protocols, and outcome reporting, a formal meta-analysis was not conducted. Instead, we synthesized the evidence into a narrative review, highlighting key findings from both preclinical and clinical spheres. The studies were grouped by:

1. Preclinical models elucidating the mechanistic underpinnings of MCT/decanoic acid actions.
2. Clinical trials and observational studies detailing dietary interventions in pediatric populations with DRE.
3. Mechanistic or structural analyses of AMPA receptor antagonism relevant to MCTs.

Emphasis was placed on pediatric-focused data to maintain clinical relevance, although select adult and veterinary studies were incorporated when they provided mechanistic insights or offered unique dietary perspectives potentially translatable to children. This approach facilitated a comprehensive overview of how MCT-based strategies, particularly decanoic acid supplementation, may influence seizure control and overall treatment feasibility in pediatric DRE. Adult studies were included only if they contributed unique mechanistic insights or offered evidence relevant to clinic application. These were clearly flagged and discussed.

**Figure 1:**
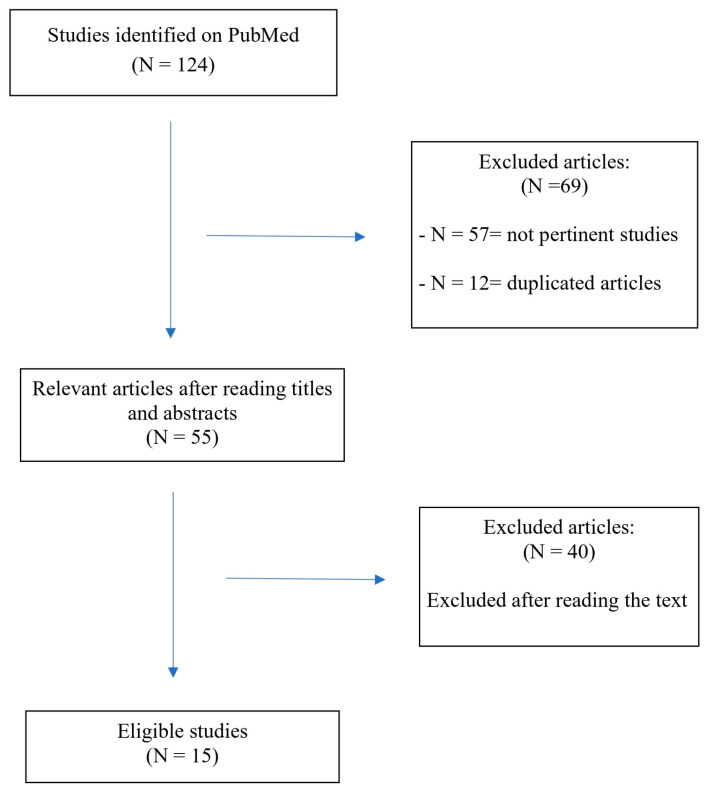
Flowchart of the search and study selection process.

In Figure 1, we report the search strategy.

## 3. Results

### 3.1. Preclinical Evidence Supporting AMPA Receptor Modulation by MCTs

- Chang et al. (2013, 2015) [9,10]:
  1. Demonstrated that MCTs (e.g., decanoic acid) reduce seizure activity in rodent models.
  2. Proposed inhibitory action at the AMPA receptor, backed by electrophysiological data.
  3. Proposed Butyl Cyclohexane Carboxylic Acid (4-BCCA), a derivative of the octanoic acid, as an alternative in cases of poor tolerability of KD, since it has shown potent antiseizure activity.

- Yelshanskaya et al. (2016) [11]:
  1. Explored structural bases of noncompetitive inhibition of AMPA-subtype ionotropic glutamate receptors by antiepileptic drugs.
  2. While not specific to MCT supplementation, the structural insights provide a mechanistic framework to understand how molecules like decanoic acid might interact with AMPA receptor sites.

- Augustin et al. (2018) [12]:
  1. Showed synergistic effects of perampanel (an AMPA receptor antagonist) and decanoic acid on seizure reduction in animal models, reinforcing the concept of decanoic acid as an AMPA receptor modulator.

- Berk et al. (2022) [13]:
  1. Although conducted in dogs with idiopathic epilepsy, this study reported metabolic shifts linked to MCT oil supplementation. The authors posited that improved seizure control could be partly derived from MCT-induced changes in neurotransmitter balance or receptor modulation.

### 3.2. Clinical Studies on MCT-Based KD

- Neal et al. (2009) [14]:
  1. Compared classical KD vs. MCT-KD in pediatric epilepsy. Both diets reduced seizures, but MCT-KD allowed for slightly higher carbohydrate intake, potentially improving dietary tolerance.

- Lambrechts et al. (2015) [15]:
  1. A prospective 2-year follow-up study in children on MCT-KD reported sustained seizure reduction. Compliance over the long term, though challenging, remained feasible for a subset of families, highlighting that MCTs might improve palatability relative to classic KD.

- Henderson et al. (2006) [16]:
  1. Meta-analysis of KD efficacy in epilepsy (not exclusively MCT-based diets), but included data supporting the potential advantages of MCT-KD in certain subgroups.

- Shin et al. (2025) [17]:
  1. Investigated a decanoic acid-enriched KD in children with refractory epilepsy. Reported notable seizure reduction in a significant proportion of participants and underscored the correlation between higher plasma decanoic acid levels and AMPA receptor modulation.

### 3.3. Trials and Observational Studies on MCT Supplementation in Less Restrictive Diets

- Borges et al. (2019) [18]:
  1. Randomized trial comparing triheptanoin vs. standard MCT oil as add-on therapy in adults with refractory epilepsy. Demonstrated partial seizure frequency reductions with MCT, offering proof of concept for less restrictive diets. Though adult-based, the rationale can be extrapolated to pediatrics.

- Schoeler et al. (2021) [19]:
  1. Investigated a blend of MCT oils as an adjunct in drug-resistant epilepsy. Showed feasibility and some seizure reduction benefits, albeit with variable individual responses. This open-label design in a less restrictive dietary context highlights the potential of partial dietary modification.

- Rasmussen et al. (2022) [20]:
  1. This preliminary study suggests that MCT oil supplementation may significantly reduce seizure frequency in adults with intractable epilepsy, with a 42% reduction observed. The intervention was generally well tolerated, with only mild gastrointestinal side effects.

The results are summarized in Table 1.

### 3.4. AMPA Receptor Modulation Mechanisms

- Chang et al. (2016) [7]
  1. Demonstrated that decanoic acid directly inhibits AMPA receptors.
  2. With an in silico docking approach, they found that the most frequent residues interacting with decanoic acid are located in the M3 helix of the transmembrane domain, which is involved in gating.
  3. They showed that non-competitive voltage- and subunit-dependent inhibition offers a distinct mechanism from ketone bodies, as decanoic acid is binding to a different site than the typical antagonists used in epilepsy treatment, such as perampanel.

- Narangoda et al. (2019) [21]
  1. Employed molecular dynamics simulations and thermodynamic integration to investigate how three structurally diverse noncompetitive inhibitors—perampanel, GYKI 53655, and CP 465022—interact with AMPA receptors.
  2. By using the crystal structures as initial binding configurations, they observed that all three inhibitors undergo several adjustments, but their binding remains stable over time.
  3. They found stable alternative binding modes to those observed in crystal structures, suggesting that the binding site is quite flexible and capable of accommodating multiple ligands in a variety of poses.

- Yelshanskaya et al. (2022) [22]
  1. Studied the interaction between the rat AMPA receptor GluA2 and 4-BCCA.
  2. Located the 4-BCCA binding sites in AMPA transmembrane domain (TMD) using X-ray crystallography, showing two 4-BCCA molecules bound in different orientations to the TMD of the channel near the pore lining M3 helices.
  3. Identified 4-BCCA binding region close to those previously predicted for decanoic acid (Chang et al. 2016 [7]), but further up in the channel and closer to the SYTANLAAF motif in M3.
  4. Showed via MD simulations and mutagenesis analysis that the 4-BCCA molecule adopts alternative orientations within its binding site; a behavior consistent with its low affinity.
  5. 4-BCCA binding is proposed to interfere with ion flow and receptor gating, hinting at possible synergy with other AMPA receptor modulators.
  6. Authors suggested that the identified 4-BCCA binding sites are probable binding regions for the whole group of medium-chain fatty acids and their branched derivatives that share the inhibitory mechanism.

**Figure 2:**
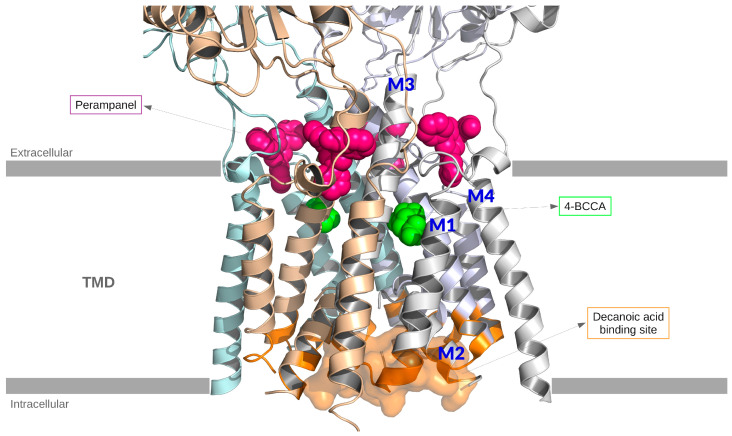
Structural representation of the tetrameric AMPA receptor crystallized with four perampanel molecules (PDB: 5l1f). For clarity, only the protein backbone is shown. Two 4-BCCA molecules have been included by aligning PDB:511f with PDB:6xsr. Four putative binding sites of decanoic acid are highlighted (orange) on the AMPA backbone. Color code: Perampanel (magenta) and 4-BCCA (green) represented by spheres, one of the binding sites of decanoic acid represented as a surface (orange). In the figure, the M1, M2, M3, and M4 helices are highlighted.

The AMPA antagonist binding modes are summarized in Figure 2.

Unlike perampanel, which binds to the TARP-associated cavity, decanoic acid interacts primarily with the M3 helix in a voltage-dependent manner, suggesting a distinct and potentially synergistic modulation strategy. While the reviewed studies offer valuable insights into MCT-based interventions and AMPA receptor modulation, it is important to recognize that much of the current evidence derives from small-scale clinical studies, preclinical animal models, and in vitro analyses. Particularly in the pediatric population, data on efficacy, safety and long-term outcomes still remain scarce. Therefore, the clinical applicability of certain findings—especially those derived from adult cohorts or experimental models—should be interpreted with caution, especially in pediatric populations.

## 4. Discussion

**Figure 3:**
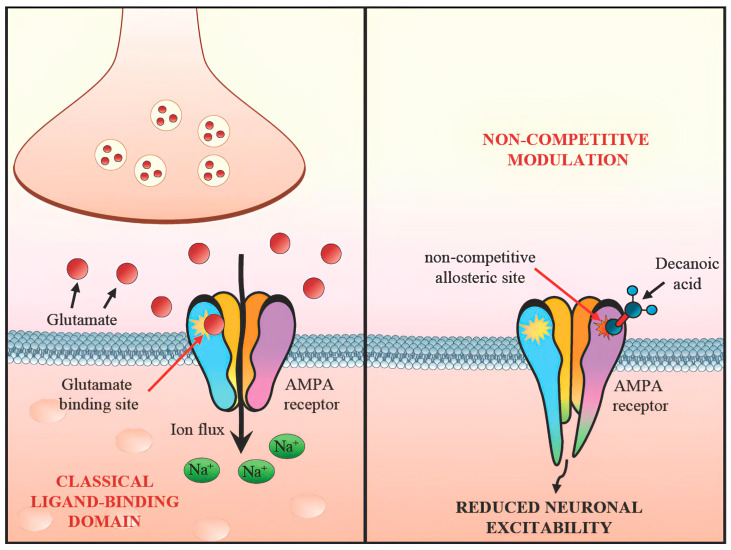
Non-competitive modulation of AMPA receptor by decanoic acid.

Recent advances in our understanding of pediatric DRE point toward the value of dietary interventions; notably, the KD and variations enriched with MCTs [23]. Central to these approaches is the recognition that seizure activity can be modulated by a range of mechanisms, from epigenetic and metabolic shifts to direct attenuation of excitatory pathways [24]. In particular, decanoic acid (C10:0), a primary component of many MCT formulations, is increasingly recognized for its ability to inhibit AMPA-type glutamate receptors in a noncompetitive fashion [25]. Studies examining the utility of MCTs have typically focused on one of two paradigms. In the first, MCTs serve as the principal fat source within a stricter KD framework (i.e., MCT-based KD), which enhances ketone body production and may optimize seizure control for those who can tolerate its stringent carbohydrate restrictions. Evidence from both Neal and colleagues (2009) [14] and Lambrechts et al. (2015) [15] emphasizes that the MCT-based KD is indeed effective, with some children experiencing long-lasting seizure reduction. These studies further suggest that, compared to the classic long-chain triglyceride (LCT) KD, the MCT-based version can occasionally permit a modestly higher carbohydrate intake, offering a marginally broader food palette. This flexibility can be beneficial for pediatric patients and their families, who often struggle with the rigidity of classical KD protocols. Nevertheless, the high level of dietary supervision, the cost of specialized MCT products, and the taste or texture issues encountered by young children all pose barriers that can limit long-term adherence. A second avenue of research explores the integration of MCTs—particularly those rich in decanoic acid—into free or less restrictive diets, aiming to capitalize on MCTs’ potential AMPA receptor modulation while mitigating the social and practical challenges inherent in a strict KD. Although this approach leads to lower ketosis, preliminary data suggest that moderate ketonemia combined with AMPA receptor modulation can still reduce seizure frequency. Initial data from pilot trials (e.g., Borges et al., 2019 [18]; Schoeler et al., 2021 [19]) in adults with drug-resistant epilepsy reveal promising but mixed outcomes, hinting that a subset of patients respond favorably to MCT supplementation without drastically altering their everyday diet. While these trials predominantly focus on adult populations in a small cohort of patients, their findings nonetheless pave the way for pediatric research, although these extrapolations to pediatric populations should be interpreted with caution due to the different pathophysiological mechanisms underlying drug-resistant epilepsies in childhood. In particular, genetic causes, which have been increasingly emerging in recent years thanks to modern genetic techniques, allow for early diagnosis with an important therapeutic prognostic implication often permitting a “tailored therapy” [24]. In this context, having a new therapeutic weapon for early use could change the natural history of many patients and their families. However, it is essential to emphasize that these studies were conducted in adults, and their findings may not fully translate to pediatric populations due to differences in metabolism, seizure etiology, and dietary compliance. Thus, extrapolation should be approached cautiously until more pediatric-specific data become available. Mechanistically, it is instructive to consider decanoic acid’s role in AMPA receptor blockade. Structural analyses (Yelshanskaya et al., 2016 [11]) highlight how molecules can engage specific noncompetitive binding sites, allowing them to reduce excitatory transmission without directly competing with glutamate. Subsequent rodent studies (Chang et al., 2013, 2015 [9,10]) illustrate that decanoic acid and related fatty acids can diminish seizure susceptibility in vivo. Augustin et al. (2018) [12] further bolster these findings by demonstrating that decanoic acid can enhance the effectiveness of perampanel, a known clinical AMPA receptor antagonist. AMPA ligands have been shown to bind to a variety of binding sites, offering potential for combination therapies (Augustin 2018, Chang 2016, Narangoda 2019, Yelshanskaya 2022 [7,12,21,22]). Taken together, these lines of evidence outline a clear rationale for why MCTs might be effective in seizure control (Figure 3). While decanoic acid exerts direct non-competitive inhibition on AMPA receptors, other mechanisms—including elevated ketone levels and anti-inflammatory effects—may act in parallel or synergistically [12,17,22]. Dissecting these effects remains a challenge due to the overlapping biochemical consequences of a ketogenic state.

Rodent models do not fully recapitulate the complexity of human epilepsies, particularly in pediatric ages and during the development of brain and neuronal connections. Therefore, translational gaps remain, and clinical validation is essential before therapeutic recommendations can be made. It is essential to recognize, however, that individual patient responses vary widely. Some children may derive substantial benefit from minimal dietary modifications and modest supplementation of MCT oil, whereas others may require more pronounced ketosis to achieve significant seizure reduction. This heterogeneity underscores the importance of ongoing research to identify biomarkers—whether genetic, metabolic, or neurophysiological—that could predict which patients are most likely to respond to a particular dietary approach. Moreover, the practical realities of implementing any specialized diet in pediatric populations, including the possibility of reduced patient compliance over time, invite additional investigations focused on acceptable taste profiles, cost-effectiveness, and caregiver education. Safety and tolerability remain paramount when introducing MCT-based therapies, especially in children. Although the majority of published pediatric data suggest that MCT consumption is generally well tolerated, it is crucial to monitor lipid profiles, gastrointestinal side effects, and potential interactions with conventional antiepileptic drugs. Long-term data on growth, bone health, and psychosocial development will further guide best practices for clinicians advising families on dietary interventions [22]. Looking ahead, larger-scale, randomized pediatric trials are needed to directly compare MCT-KD with a free diet plus MCT supplementation and elucidate whether there is a clinically significant difference in seizure outcomes, growth parameters, and overall quality of life. Such studies would ideally incorporate neuroimaging, metabolic profiling, and advanced electrophysiological assessments to capture the full spectrum of therapeutic effects [26]. Long-term MCT administration requires monitoring for growth retardation, gastrointestinal intolerance, and lipid abnormalities. Periodic assessments of BMI, liver function, and developmental milestones are advised in children on MCT-enriched regimens [14]. 

By doing so, we can move closer to effective, sustainable, and family-friendly dietary interventions that meaningfully improve the lives of children with drug-resistant epilepsy. In Table 2, we summarize the main differences between classic KD and MCT supplementation. In Table 3, we report the clinical use of MCTs including dosage, safety, and adherence guidance.

Strategies to improve adherence include using flavored or emulsified MCT oils, involving dietitians in meal planning, and structured caregiver education programs. Behavioral support also plays a key role in maintaining dietary compliance over time [27].

## 5. Conclusions

DRE requires, especially in pediatric age, multifaceted management strategies that transcend standard pharmacotherapy. A MCT-based KD presents a promising avenue, leveraging robust ketosis and direct receptor-level modulation (particularly AMPA receptor antagonism via decanoic acid). However, the adherence challenges of a classic KD remain a significant barrier for many families. Emerging evidence suggests that add-on MCT supplementation within a less restrictive diet may mitigate these challenges, offering partial but clinically meaningful seizure control in some children. Further pediatric-specific trials are essential to validate MCT supplementation as a scalable and tolerable adjunct for families unable to commit to full ketogenic protocols.

## 6. Limits

This review presents promising evidence supporting the role of MCT-based dietary interventions, particularly those involving decanoic acid, in managing drug-resistant epilepsy (DRE). However, several limitations must be acknowledged:

1. Study heterogeneity: The included studies varied significantly in design, population (pediatric vs. adult), MCT formulations, dosing regimens, and outcome measures. This heterogeneity precluded formal meta-analysis and limits the generalizability of the findings.

2. Pediatric extrapolation from adult data: Some mechanistic and clinical insights were derived from adult studies (e.g., Borges et al., Schoeler et al.), which may not fully translate to pediatric populations due to differences in metabolism, seizure etiology, and dietary tolerance. While these were included to illustrate potential mechanisms, caution is warranted when interpreting their clinical relevance to children.

3. Limited sample sizes and open-label designs: Many of the included clinical studies, particularly those specific to MCT supplementation, were small-scale (e.g., Shin et al., *n* = 15) or lacked blinding, increasing the risk of bias. These factors reduce the strength of the evidence and highlight the need for larger, well-controlled pediatric trials.

4. Scarcity of long-term data: Few studies addressed long-term outcomes such as growth trajectories, bone health, cognitive development, or psychosocial impacts of sustained MCT use in children. This represents a critical gap given the vulnerability of pediatric populations.

5. Mechanistic complexity: While AMPA receptor inhibition by decanoic acid is a key mechanistic focus, other potential pathways (e.g., anti-inflammatory, mitochondrial, epigenetic) may contribute to seizure control. These were not systematically dissected across studies, limiting mechanistic precision.

6. Potential selection and publication bias: As a narrative review with a systematic search component, there remains an inherent risk of selection bias. The predominance of positive studies may reflect publication bias, and negative or null results may be underrepresented.

7. Structural Interpretation Limits: Although molecular docking and crystallography data suggest plausible binding interactions between MCTs and AMPA receptors, most findings remain preclinical. Structural analyses, while informative, require in vivo validation before conclusions can be drawn about efficacy or safety in children.

In light of these limitations, further pediatric-specific research is urgently needed to validate the therapeutic role of MCTs, optimize dosing strategies, and ensure safe long-term use in children with drug-resistant epilepsy.

## Figures and Tables

**Table 1:** Selected preclinical and clinical studies on MCT-based or MCT-supplemented diets for epilepsy.

Author/Year	Study Type	Population/Model	Diet/Intervention	Key Outcomes	AMPA Receptor Link	Limitations	MCT Dosage	Ketone Levels	Side Effect
Shin, 2025 [17]	Clinical (prospective)	Pediatric refractory epilepsy (*n* = 15)	Decanoic acid enriched KD	Reduced seizures in a significant subset. High decanoic acid levels correlate with improved seizure control.	Direct: Decanoic acid shown to inhibit AMPA receptors	High cost; potential poor adherence in some families	4 g/100 mL	NA	Transient hypoglycemia and metabolic acidosis; hypercalciuria; transient gastrointestinal symptoms
Chang, 2013 [9]	Preclinical (rodent)	Rodent seizure model	MCT supplementation (lab diet)	Reduced seizure susceptibility in rodents Identified MCFAs’ capacity to modulate excitability	Noncompetitive inhibition of AMPA receptors	Animal study; translation to humans requires caution	100 mM	NA	Sedaction
Chang, 2015 [10]	Preclinical (rodent)	Rodent seizure model	MCFA derivatives	Enhanced potency against seizures through chain structure modifications	Supports decanoic acid as partial AMPA antagonist	Synthetic derivatives may differ from dietary MCT formulations	100–125 mg/kg	NA	Teratogenicity
Yelshanskaya, 2016 [11]	Structural in vitro	Human/rodent AMPA receptors (in vitro)	Drug binding studies (AMPA receptor)	Clarified architecture of noncompetitive inhibition; provided insight into how molecules (including decanoic acid analogs) may bind	Mechanistic blueprint for decanoic acid activity	Not an in vivo or dietary study	NA	NA	NA
Neal, 2009 [14]	Clinical (RCT)	Children with epilepsy (*n* = 145)	Classical KD vs. MCT-KD	Comparable seizure reduction; MCT-KD slightly more flexible with carbs	MCT-KD hypothesized to modulate AMPA via decanoic acid production	Adherence challenges; short to mid-term follow-up	started at 40–45% of energy, increased up to 60% if necessary	0.696 ± 0.475 mmol/L (3 months)	Gastrointestinal
Lambrechts, 2015 [15]	Clinical (prospective)	Children with refractory epilepsy (2-year follow-up)	MCT-KD	Sustained seizure reduction; better tolerability vs. classic KD in some patients	Likely partial AMPA receptor modulation via decanoic acid	Non-randomized; potential selection bias	10 g/die	3.1 mmol/L (12 months)	Gastrointestinal, fatigue
Augustin, 2018 [12]	Preclinical (rodent)	Rodent seizure model and in vitro assays	Perampanel and decanoic acid	Synergistic anticonvulsant effect; enhanced blockade of AMPA receptors	Confirmed synergy with known AMPA antagonist (perampanel)	Lack of direct translational data to pediatric populations	~157 μmol/L (plasma)	NA	NA
Henderson, 2006 [16]	Meta-analysis	Pediatric KD studies (various designs)	Classical KD and MCT-KD	KD (including MCT-KD) shown to be effective across multiple studies	Potential mechanism includes AMPA modulation by decanoic acid	Heterogeneous study designs; older references	NA	NA	Gastrointestinal, weight loss, irritability, increased serum cholesterol or triglyceride and liver anzymes, lethargy, hypercalciuria, renal stones, hypoglicemia
Berk, 2022 [13]	Clinical (observational)	Dogs with idiopathic epilepsy	Free diet + MCT	Reduced seizure frequency; metabolic fingerprints	Possibly relevant to AMPA receptors, but not directly tested	Veterinary study; extrapolation to humans limited	9% of metabolic energy	0.06 mmol/L	Not reported
Borges, 2019 [18]	Clinical (RCT)	Adults with DRE	Add-on triheptanoin vs. MCT	Demonstrated partial seizure frequency reduction; proof-of-concept for less restrictive approach	Decanoic acid among possible MCFA mediators	Adult population; short follow-up	35% of energy	NA	Gastrointestinal, headache, disturbed sleep pattern
Schoeler, 2021 [19]	Clinical (feasibility)	Adults and adolescents with DRE	MCT as add-on	Feasibility and partial seizure control benefits; high inter-individual variability	Potential for AMPA receptor modulation via C10:0 and related MCFA	Non-randomized; open-label design	Adult: 240 mL; children: 19% daily energy (120 mL)	>1 mmol	Gastrointestinal
Rasmussen, 2022 [20]	Clinical (RCT)	Adults with DRE	Free diet + MCT	Seizure reduction	Potential mechanism includes AMPA modulation	Adult population, small cohort	15 mL up to 60 mL daily (14 g of MCT/15 mL)	Presence in urine	Gastrointestinal

Abbreviations: KD = Ketogenic Diet; MCT = Medium-Chain Triglycerides; MCFA = Medium-Chain Fatty Acids; RCT = Randomized Controlled Trial; DRE = Drug-Resistant Epilepsy.

**Table 2:** Comparison between classic KD and MCT supplementation approaches.

Feature	Classic Ketogenic Diet	MCT Supplementation
Efficacy	High in selected patients; often requires sustained ketosis	Moderate; some benefit even with low ketosis
Dietary flexibility	Very restrictive; low carbohydrate allowance	Less restrictive; more flexible food choices
Palatability	May be challenging for children	Improved palatability with oils or emulsion
Tolerability	Gastrointestinal side effects common	Generally well tolerated, but varies by dose
Adherence	Difficult to maintain long-term	Potentially better due to lower restriction

MCT = medium-chain triglycerides.

**Table 3:** Clinical use of MCTs: dosage, safety, and adherence guidance.

Aspect	Recommendation/Findings
MCT dosage (pediatric)	0.5–1.5 g/kg/day (divided dose), based on tolerance and metabolic response
Duration	Minimum 3 months for efficacy assessment; some protocols extend to 12+ months
Administration form	Emulsified oil, flavored liquids, or incorporated into meals/snack
Common side effects	Gastrointestinal discomfort (bloating, diarrhea), lipid profile alterations
Monitoring parameters	Growth charts (weight, height), lipid panels, liver enzymes, ketone levels
Adherence tips	Use of palatable formulations, caregiver education, involvement of dietitians
Contraindications	Fat metabolism disorders, pancreatitis, certain liver diseases
Co-therapies	Can be combined with AEDs; interactions generally minimal, but monitoring is advised
Follow-up frequency	Monthly in the first 3 months; every 3–6 months thereafter

## Data Availability

The original contributions presented in the study are included in the article, further inquiries can be directed to the corresponding author.
